# First Phylodynamic Analysis of HRSV‐A and HRSV‐B in Rio de Janeiro, Brazil, Reveals Recurrent Dispersals From the Megalopolis Center to Suburban Cities

**DOI:** 10.1002/jmv.71177

**Published:** 2026-10-15

**Authors:** Alessandra Pavan Lamarca, Melise Chaves Silveira, Renata Campos Azevedo, Maria Angélica Arpon Marandino Guimarães, Claudia Maria Braga de Mello, Adriana Cardoso Camargo, Leandro Magalhães de Souza, Alexandra Lehmkuhl Gerber, Ana Paula C. Guimarães, Andréa Cony Cavalcanti, Ana Tereza Ribeiro Vasconcelos

**Affiliations:** ^1^ Campus Duque de Caxias, Universidade Federal Do Rio de Janeiro Rio de Janeiro Brazil; ^2^ Laboratório de Bioinformática. Laboratório Nacional de Computação Científica (LNCC/MCTI) Rio de Janeiro Brazil; ^3^ Departamento de Virologia, Instituto de Microbiologia Paulo de Góes, Universidade Federal do Rio de Janeiro Rio de Janeiro Brazil; ^4^ Departamento de Doenças Infecciosas e Parasitárias Faculdade de Medicina, Hospital Universitário Clementino Fraga Filho, Universidade Federal do Rio de Janeiro Rio de Janeiro Brazil; ^5^ Laboratório Central de Saúde Pública do Estado do Rio de Janeiro Rio de Janeiro Brasil; ^6^ Secretaria de Saúde do Estado do Rio de Janeiro Rio de Janeiro Brasil

**Keywords:** coinfection, genomic surveillance, *Orthopneumovirus hominis*, phylodynamics, spatial transmission

## Abstract

Human respiratory syncytial virus (HRSV) is the leading cause of acute lower respiratory tract infections in infants and represents a major burden to public health systems worldwide. Despite its epidemiological relevance, genomic surveillance of HRSV in Brazil remains limited. Here, we investigated the genetic diversity and dispersal dynamics of HRSV‐A and HRSV‐B in the state of Rio de Janeiro, Brazil, during the period immediately preceding the implementation of maternal vaccination and prophylactic monoclonal antibodies. A total of 136 near‐complete HRSV genomes were recovered from respiratory samples collected between May 2024 and June 2025 using metagenomic sequencing. Phylogenetic analyses identified 11 HRSV‐A and five HRSV‐B lineages, with most sequences belonging to the A.D.3 and B.D.E.1 clades. Phylodynamic reconstructions revealed multiple independent introductions into the state, with the city of Rio de Janeiro acting as the primary within‐state dispersal hub. Viral dispersal routes originating from the capital also showed greater mean transmission distances than those from other municipalities. Coinfections with additional respiratory viruses were frequently detected, and most HRSV‐positive patients were children under 1 year of age. These results demonstrate that HRSV circulation in Rio de Janeiro is shaped by repeated introductions and strong spatial structuring centered around highly connected urban areas. Our findings underscore the importance of continuous genomic surveillance to monitor viral evolution, characterize transmission dynamics, and support science‐based public health strategies against HRSV. Establishing robust monitoring systems will be critical to evaluate the impact of newly implemented immunization programs in Brazil.

## 1 Introduction

The human respiratory syncytial virus *Orthopneumovirus hominis* (HRSV) is an enveloped, negative‐sense, single‐stranded, 15.2 kb RNA virus belonging to the family *Pneumoviridae*. It is recognized as one of the leading pathogens of the human lower respiratory tract, causing acute bronchiolitis and pneumonia with hypoxemia. The species is classified into two antigenic groups, HRSV‐A and HRSV‐B, that are distinguished primarily by mutations in their G gene [1]. The circulation dynamics of both viruses are often characterized by one subgroup predominating during a given epidemic season while the other persists at lower frequencies [2, 3]. This is a consequence of the partial cross‐protection between HRSV‐A and HRSV‐B, although this immunological response is probably insufficient to prevent reinfection [1]. It has also been suggested that HRSV‐A infections are associated with greater clinical severity than HRSV‐B infections, but no consensus has been drawn [3, 4].

Although HRSV infection is generally mild in healthy adults, it constitutes a major cause of hospitalization in early childhood, particularly in the first year of life. Global burden estimates indicate that almost 2.2 million children require hospital admission due to HRSV‐associated lower respiratory diseases before turning one [5]. Infants under 6 months of age are disproportionately affected, accounting for 51% of the more than 26 300 HRSV‐attributed annual deaths of children under 5 years old. The burden of HRSV is also not evenly distributed across global regions and nearly 95% of HRSV‐associated deaths occur in middle‐ and lower‐income countries.

In a 19‐year period, more than 600 000 hospitalizations of children under 12 months happened in Brazil due to acute viral bronchiolitis, with HRSV possibly causing half of this number [6, 7]. Although HRSV‐associated fatalities are reduced, each hospitalization costs more than USD $5900 annually, adding a high economic burden on the Brazilian public health system [8]. In December 2025, Brazil started the maternal vaccination program against HRSV. Evidence has shown that this vaccine could reduce in 72% neonatal hospitalization due to HRSV and in more than 60% the development of lower respiratory diseases in infants under 6 months [9, 10]. The monoclonal antibody nirsevimab demonstrates an effectiveness of approximately 80% in preventing HRSV‐associated hospitalization [11, 12] and was also approved for prophylactic use in Brazil in February 2026.

The Brazilian state of Rio de Janeiro has an annual incidence of ~13 hospitalizations due to bronchiolitis per 1000 infants under 1 year, with at least half of these attributed to HRSV infections [6, 7, 13]. Being home to more than 16 million people, the state has an extremely heterogeneous population density, with cities ranging from 12 500 hab/km^2^ (São João de Meriti) to 12 hab/km^2^ (Santa Maria Madalena) [14]. As population density directly influences the risk of local outbreaks of airborne vectors and delimits the immediate availability of primary care clinics and hospitals [15], this complex landscape generates a logistical challenge in how to better utilize the scarce resources allocated for vaccine and monoclonal antibody purchase and distribution.

Analyses of viral genomic sequences can detail the spatial dynamics that determine how HRSV spreads and evolves. By identifying circulating viral lineages and newly introduced variants, it is possible to distinguish continuous local transmission from repeated new introductions, identifying areas that act as sources of viral dissemination. This approach is particularly valuable for public health planning, as it allows authorities to identify locations and periods of higher transmission risk and plan science‐informed interventions. In addition, monitoring of viral genetic diversity enables the early detection of mutations that may affect viral fitness or reduce the effectiveness of immunization strategies, ensuring that prevention programs remain effective while HRSV continues to evolve [12, 16].

The present work is the first step toward building a genomic surveillance framework for HRSV in the state of Rio de Janeiro, Brazil. It investigates the genetic diversity of both HRSV‐A and HRSV‐B in the state in a period just prior to the approval of maternal vaccination and use of prophylactic monoclonal antibodies. With the genomic sequences obtained, we also evaluate the geographical dynamics of within‐state viral dispersal by reconstructing the routes by which new HRSV genotypes are spreading across Rio de Janeiro. Finally, we also comment on the age structure of patients infected with HRSV and on biases in the current surveillance system.

## 2 Methods

### 2.1 Sampling and Library Preparation

The Noel Nutels Central Public Health Laboratory of the state of Rio de Janeiro (LACEN‐RJ) routinely receives biological samples taken from human patients treated within the state's public health system. Reasons for sampling can range from routine surveillance of respiratory diseases to the diagnosis of critically ill patients. For this observational retrospective work, we randomly selected 472 samples, collected from symptomatic patients between May 2024 and June 2025. Demographic and clinical data were obtained from laboratory records and, when available, from associated health information systems. The main variables included patient age, sex, geographic origin, and sample type. Due to the study's retrospective design, the completeness of clinical data varied across samples. The present work was approved by the Ethics Committee of Universidade Federal do Rio de Janeiro (Process number 7.686.303).

Total nucleic acids were extracted using the MagMAX Viral/Pathogen Nucleic Acid Isolation Kit (Thermo Fisher Scientific) with an input volume of 200 µL and a final elution volume of 60 µL. We employed the Viral Surveillance Panel v2 (Illumina), which targets more than 200 RNA and DNA viruses, to conduct whole‐genome amplification and library preparation of the extractions. Library concentration and integrity were determined using the Agilent TapeStation 4200 System (Agilent Technologies, USA) according to the manufacturer's instructions.

### 2.2 Sequencing and Assembling

Libraries were sequenced using the Illumina NextSeq 2000 platform (Illumina, San Diego, CA, USA), generating 150‐bp paired‐end reads. Quality control was conducted with fastp [17] using default parameters. We mapped reads to the human reference genome (GRCh39.p14: GCA_000001405.29) and obtained the unmapped reads from this alignment using the SAMtools package [18]. To extract the viral data contained within the unmapped reads, we mapped these to a reference dataset composed of the viral genomes indicated in the DRAGEN Microbial Enrichment Plus protocol in addition to two human rhinovirus genomes (MF043119.1 and NC_038878.1). Variant calling was then conducted using bcftools [18] (bcftools mpileup ‐E ‐Ou and bcftools call ‐‐ploidy 1 ‐mv), and the consensus genome was built using BedTools2 [19] (bedtools genomecov ‐bga ‐ibam and masked as N using bcftools consensus ‐‐mask). Genome quality and completeness are described in Table S1. We considered a sample positive for a specific virus when its genomic coverage was above 98%.

### 2.3 Phylogenetic Analyses

In order to describe the genotypic diversity in our samples, we used MAFFT 7 [20] to align the new HRSV‐A and HRSV‐B sequences against the reference genomes made available by the HRSV Genotyping Consensus Consortium [21] (https://github.com/rsv-lineages). The resulting alignment was used to construct a phylogenetic tree for each virus using IQ‐TREE 2 [22] with the GTR+F+I+G4 model in both runs, selected by the ModelFinder algorithm [23] built within IQ‐TREE. The resulting tree was then used to classify each new sequence into lineages following the basis proposed by Goya et al. (2024). Lineage assignment was also conducted through the Nextclade application version 3.20.0 [24].

As A.D.3 was the most common HRSV‐A lineage in our dataset (See Section 3), we obtained all viral genomes available in the Pathoplexus database with complete sample collection dates and sequence lengths greater than 12 000 bp that belonged to this clade. These genomes were included primarily to provide broad phylogenetic context for the newly generated sequences and to minimize the risk of artificially grouping distinct clades occurring within Rio de Janeiro due to insufficient sampling of closely related viruses in the background datasets. This allowed us to infer the minimum number of introductions of RSV‐A in the state. The background sequences were aligned to the new Brazilian sequences, and this final dataset was used to infer a new phylogeny with IQ‐TREE. We reconstructed ancestral geographic locations using the “mugration” model available in TreeTime [25] and the geographic locality of each sampled genome was treated as a discrete character. Changes in the inferred ancestral state along the phylogeny were used to identify geographic transitions between localities, which were interpreted as viral dispersals. The same analysis was repeated with the HRSV‐B lineage B.D.E.1. Finally, we calculated the mean dispersal distance using the cities' geographical centroids, calculated with the R package *sf* and the shapefiles provided by the Instituto Brasileiro de Geografia e Estatística (IBGE, https://www.ibge.gov.br/geociencias/organizacao-do-territorio/malhas-territoriais/15774-malhas.html).

To evaluate the influence of sampling bias in our results, we estimated the expected number of sequences from each Rio de Janeiro municipality based on population size from the 2022 Census [14] and the official number of SARS cases in the state, obtained from the Epidemiological Surveillance Information System for Influenza (SIVEP‐Gripe), a public database of SARS cases maintained by federal and state governments in Brazil [26]. We removed terminal branches from A.D.3 and B.D.E.1 phylogenetic trees that belonged to oversampled locations until these reached the expected sample sizes, which were determined through the comparison of Gini Coefficients of Intensity. We kept a minimum of one sample per location. We ran 50 replicates for each RSV subgroup and estimated the number of introductions to Rio de Janeiro and dispersals originating in each city.

## 3 Results

The 472 sequenced samples originated from investigations of critically ill patients with respiratory symptoms (*n* = 332) and from surveillance testing in symptomatic patients (124), although 15 samples did not have this information (Figure 1A; Table S2). Most of the samples (*n* = 196) with patient age information were of children aged 5 or below (Figure 1A,B). A total of 136 samples had sequence reads that matched more than 98% of HRSV‐A (*n* = 82) and RVA‐B (*n* = 52) reference genomes. The cohort of patients positive for HRSV‐A was composed of 31 females, 43 males, and 7 people with undisclosed sex. All the patients were children under 5 years (Figure 1C), except for one male older than 60 years. Two deaths were reported among these patients: of the aforementioned 60+ adult, who also had malignant neoplasm of the peritoneum; and of a 3‐month‐old male infant without comorbidities. The 55 HRSV‐B patients were composed of 20 females and 34 males. Once again, all cases were of children under 5 years old (Figure 1D), apart from an adult male younger than 60 years old. A single death of a 1‐month‐old male baby with Klinefelter Syndrome was registered.

**Figure 1:**
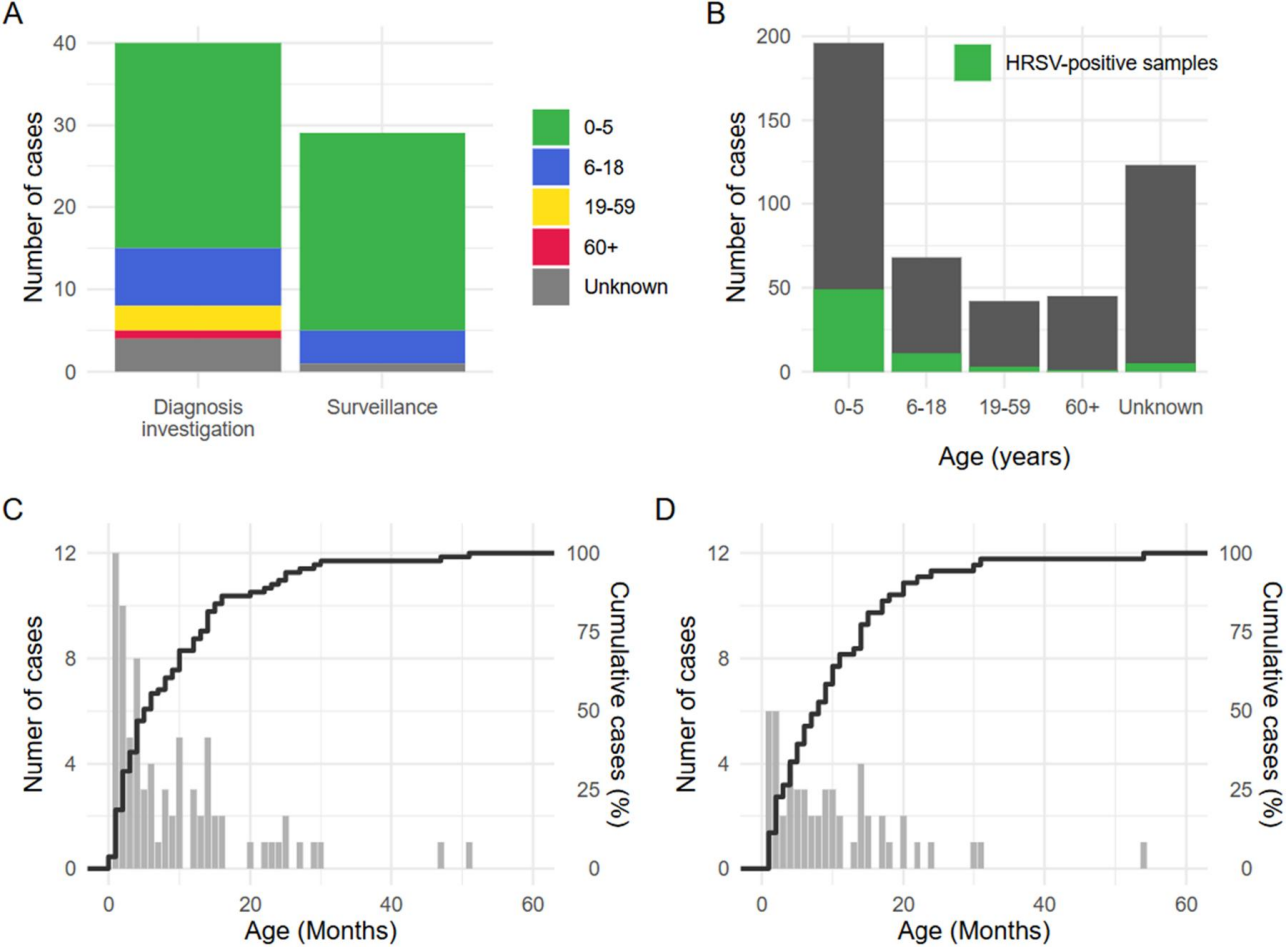
Age structure of HRSV‐positive cases in our sample. In (A), HRSV‐positive samples are classified by the reason for sampling and by patient age. In (B), the whole dataset is represented in gray, while HRSV‐positive cases are colored in green. The number of children under 5 years old positive for HRSV‐A and HRSV‐B, and their cumulative sums, are represented respectively in (C) and (D).

A total of 26 patients also had more than 98% genomic coverage for a second virus, all of them associated with respiratory diseases (Table 1). Lowering this threshold to 90% and to 80% of genomic coverage resulted in 50 and 69 patients with co‐detections, respectively (Table S3). Three of the 98%‐coverage co‐detections were of HRSV‐A with HRSV‐B, with one case also positive for a third virus, human adenovirus E. Human adenoviruses were the ones most commonly found among the 98%‐coverage coinfections, followed by human parainfluenza and bocaviruses. One of the HRSV‐A and human bocavirus co‐detection cases was that of the 3‐month‐old infant who died.

**Table 1:** List of viruses present in the 98%‐coverage co‐detection cases.

Human respiratory syncytial virus	Coinfection virus	Number of samples
HRSV‐A	HRSV‐B	3
HRSV‐A	Human adenovirus E	3
HRSV‐A	Human bocavirus	2
HRSV‐A	Human coronavirus OC43	2
HRSV‐A	Human parainfluenza type 1	2
HRSV‐A	Human parainfluenza type 2	1
HRSV‐A	Human parainfluenza type 3	2
HRSV‐A	Influenza A subtype H1N1	1
HRSV‐A	Influenza C	1
HRSV‐A	Rhinovirus A	2
HRSV‐A	SARS‐Cov‐2	1
HRSV‐B	Human adenovirus C	1
HRSV‐B	Human adenovirus D	2
HRSV‐B	Human adenovirus E	1
HRSV‐B	Human bocavirus	2
HRSV‐B	Human coronavirus 229E	1
HRSV‐B	KI polyomavirus	1
HRSV‐B	MW polyomavirus	1
HRSV‐B	Rhinovirus A	1

The HRSV‐A genomes were distributed across 11 lineages based on their phylogenetic positioning relative to the reference genomes, while Nextclade identified nine lineages (Figure 2A). In both, A.D.3.3 was the most common lineage, and the whole A.D.3 clade (including sublineages) comprised 83% of samples. The HRSV‐B, on the other hand, had lower diversity in our samples, with five lineages present according to their phylogenetic positioning and only three lineages present according to the Nextclade analysis (Figure 2B). The lineage B.D.E.1 was the most common in both classifications (39 and 43 samples, respectively), and the whole clade composed more than 81% of samples.

**Figure 2:**
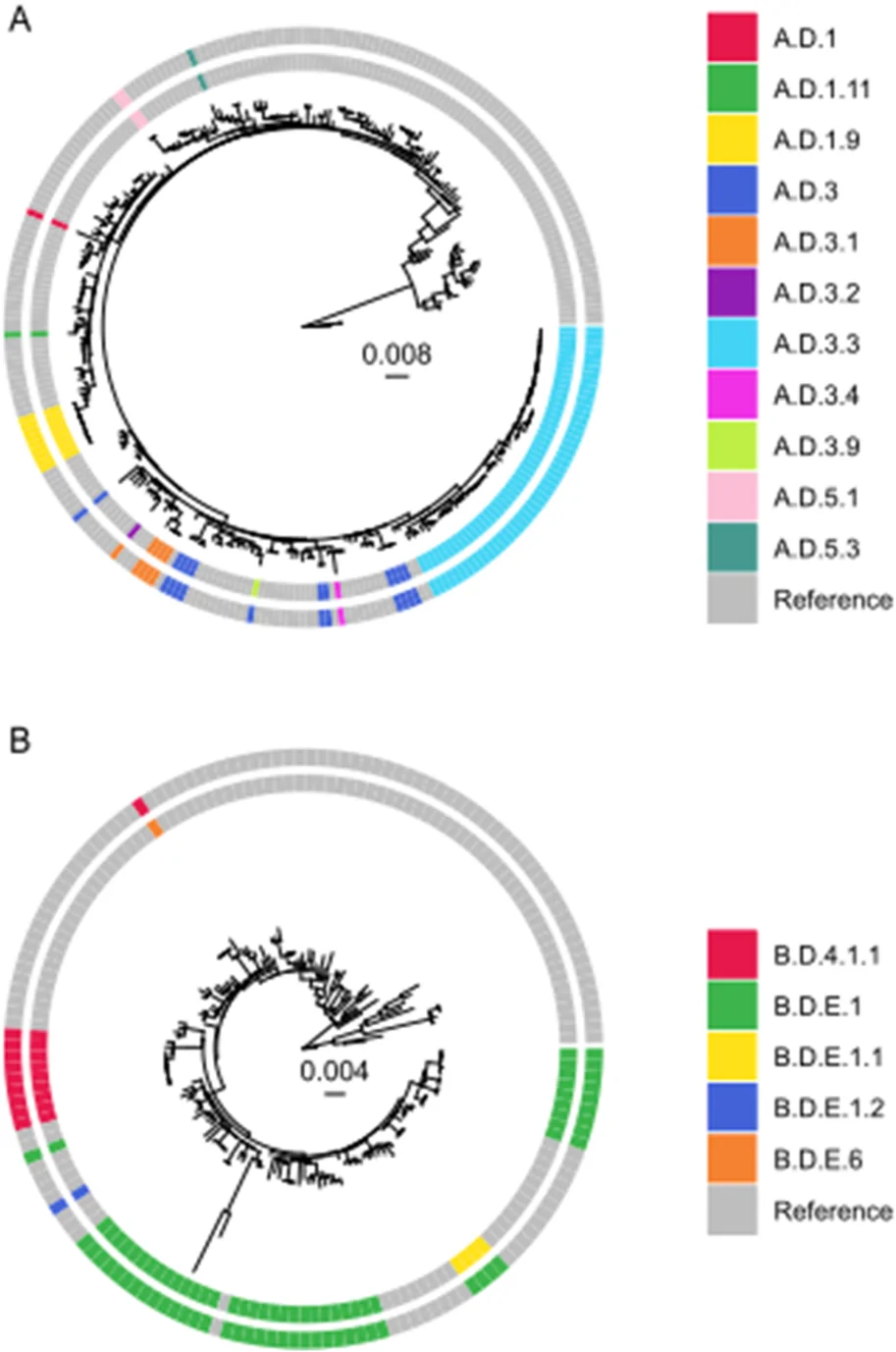
Evolutionary position of the newly sequenced HRSV‐A (A) and HRSV‐B (B) genomes in comparison to the references made available by the HRSV Genotyping Consensus Consortium. In both trees, the inner ring represents the lineages defined by their phylogenetic position, while the outer ring represents their Nextclade classification. Reference sequences are represented in gray.

As A.D.3 was the most common HRSV‐A clade in our analysis, we used it as a model to further investigate the HRSV dispersal dynamics within the state of Rio de Janeiro. To accomplish this, we generated a new phylogeny using 2306 additional HRSV‐A background genomic sequences. Based on these inferences, we count at least 11 introductions of the A.D.3 clade in the state (Table S1). Subsampling the Brazilian sequences so they reflect the cities' population sizes resulted in a mean of 9.46 introductions (s.d. = 0.93), while guiding this subsampling by the official number of SARS cases in the state of Rio de Janeiro indicates a mean of 9.76 introductions (s.d. = 0.85). A single Brazilian sequence present in the background dataset was not directly related to any of our samples and was considered another independent introduction to the country. Imports of A.D.3 were inferred to have happened through the cities of Duque de Caxias (1), Nova Iguaçu (1), Rio de Janeiro (7), and São Gonçalo (2). Despite the considerable number of entrance routes, within‐state dispersal was categorized by the city of Rio de Janeiro acting as the main source of transmission (Figure 3). The importance of the city of Rio de Janeiro is consistent through the subsampled replicates, although we observe variability in the participation of smaller cities in the dispersal dynamics in the state (Table S4). For the A.D.3 clade, mean dispersal distances were 69.18 km for routes starting in the city of Rio de Janeiro, and 45.34 km for the other routes.

**Figure 3:**
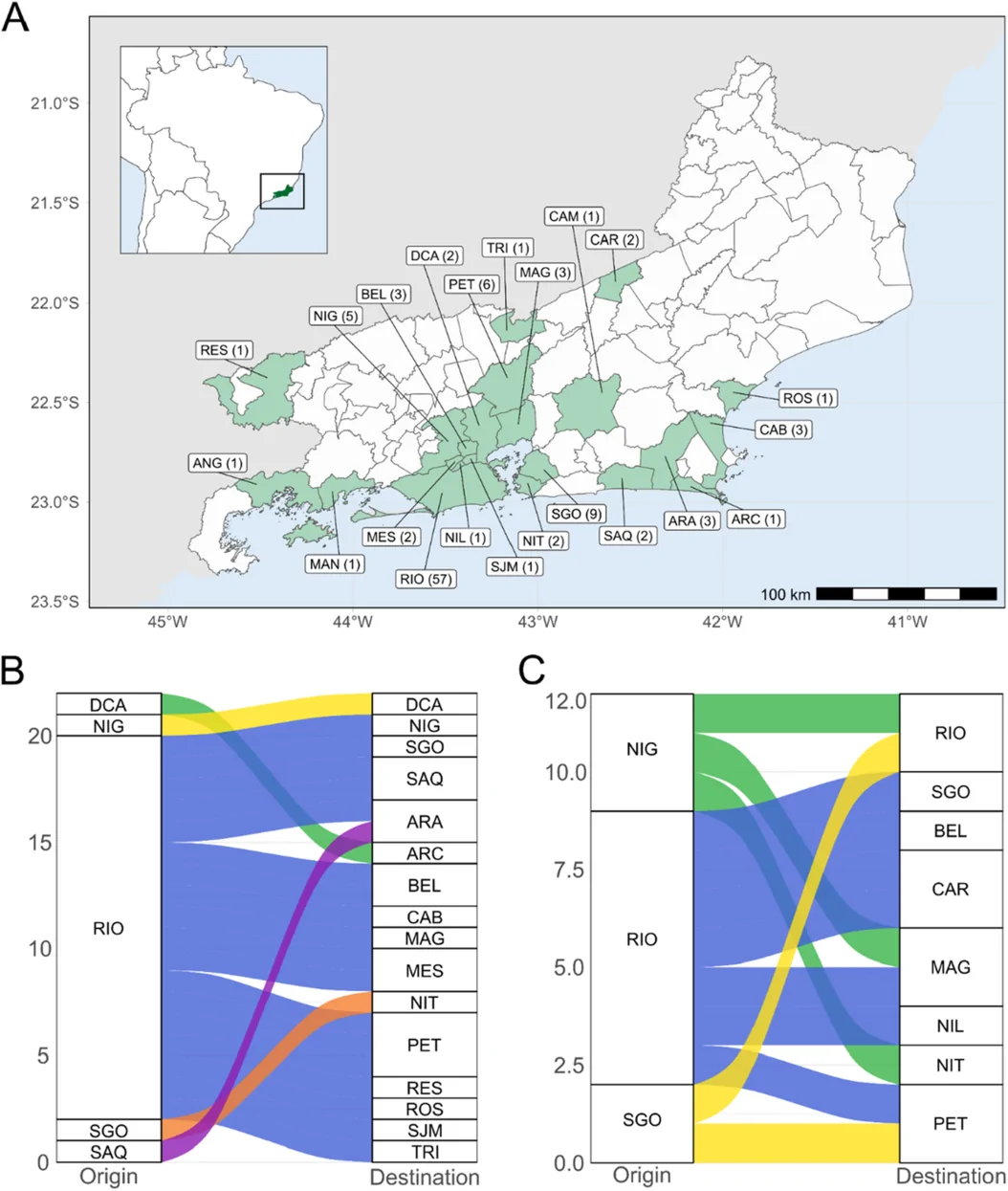
Map of the state of Rio de Janeiro (A) and the number of dispersals between its cities inferred for the A.D.3 clade (B) and the B.D.E.1 clade (C). In the map, cities with HRSV sequences are colored in green, and the number of said sequences is shown in parentheses. On the right of figures (B) and (C) are the sources of dispersal, while on the left are the destinations. Colors indicate the same source of dispersal. Abbreviations indicate the corresponding city: ANG = Angra dos Reis; ARA = Araruama; ARC = Arraial do Cabo; BEL = Belford Roxo; CAB = Cabo Frio; CAM = Cachoeira de Macacu; CAR = Carmo; DCA = Duque de Caxias; MAG = Magé MAN = Mangaratiba; MES = Mesquita; NIL = Nilópolis; NIT = Niterói; NIG = Nova Iguaçu; PET = Petrópolis; RES = Resende; ROS = Rio das Ostras; RIO = Rio de Janeiro (state capital); SGO = São Gonçalo; SJM = São João de Meriti; SAQ = Saquarema; TRI = Três Rios.

We conducted the same analysis for clade B.D.E.1, as it was the most common HRSV‐B lineage in our dataset. In addition to the newly sequenced genomes, we collected 5053 background genomic sequences and inferred a new phylogeny (Table S1). We observed at least 21 introductions to the state of Rio de Janeiro, with a mean of 19 introductions (s.d. = 0.83) in the replicates subsampled by population size and 19.16 introductions (0.95) in the subsamples by number of SARS cases. Unfortunately, there were no other Brazilian sequences in the background dataset that passed through our filtering requirements, so we could not evaluate the dispersal of this clade to other states. B.D.E.1 imports occurred through the cities of Angra dos Reis (1), Araruama (1), Cabo Frio (1), Mangaratiba (1), Nova Iguaçu (1), Petrópolis (1), Rio de Janeiro (13) and São Gonçalo (2), with mean dispersal distances of 69.86 km from routes starting in Rio de Janeiro and 43.59 km for routes starting in other cities. Again, the large participation of the city of Rio de Janeiro was corroborated by the replicates of both subsampling strategies used (Table S4).

## 4 Discussion

The present study has, for the first time, analyzed, under a phylodynamic scope, the dispersal of HRSV‐A and HRSV‐B in the state of Rio de Janeiro, Brazil. While we have detected limited viral diversity circulating in the state, the A.D.3.3 and B.D.E.1 genotypes encountered result from multiple introductions happening mostly through the city of Rio de Janeiro, the largest tourist hub in the country. Within the state, we observed that these lineages spread 20 km further when originating in its homonymous capital when compared to when leaving from other cities.

Beyond being the second largest city by population in Brazil, Rio de Janeiro had 12.5 million visitors in 2025, with 2.1 million of them being foreigners. The large flow of people in tourist cities increases the risk of importing new viral strains, with said cities becoming dispersal hubs to neighbors, which enables local outbreaks [27]. It is clear from our data that the city of Rio de Janeiro plays this role, most commonly being the genealogical origin of the samples collected across the state. Similar results have been observed for both the city and the state of Rio de Janeiro in the dispersal of SARS‐CoV‐2 [28] and arboviruses [29].

These findings reinforce the importance of pathogen surveillance in regions with high international traffic. Efficient random testing in airports may prove itself unfeasible, and tracking ill tourists nearly impossible. But testing returning travelers that have fallen ill could provide an effective sentinel system, as shown by different initiatives [30, 31]. Brazil's public healthcare system is composed of a nation‐wide network of free‐attendance hospitals and primary care clinics, with mandatory reports for several diseases, such as COVID‐19 and tuberculosis (but not HRSV). Such accessible and broad healthcare coverage would permit establishing a surveillance protocol for sequencing samples from patients who have recently traveled. The main challenges for such implementation lie in the limited funding available for genomic sequencing and the large distance between hospital/clinics, the central laboratories for public health (LACEN), and the sequencing facilities. But we reinforce that the costs for treating HRSV patients, especially those under 5 years old, surpass those of genomic surveillance [8, 32, 33, 34].

The HRSV samples here sequenced were grouped into 16 different evolutionary lineages, following the classification proposed by Goya et al. (2024). The A.D.3.3 lineage corresponded to 61% of our HRSV‐A sequences. Previous studies reported A.D.3.3 occurring at low frequencies (≤ 3%) in Europe and South America through the 2021–2025 season [35, 36, 37]. GISAID's dashboard reports A.D.3.3 worldwide rates ranging from 1% (September 2024) to 14% (June 2024) in the period encompassed by our work. When considering only data from the Americas, A.D.3.3 frequencies ranged from 3.2 (October 2024) to 29% (June 2025) in the same period. In this smaller dataset, the lineage seems to increase annually between April and July, subsiding thereafter. This coincides with the observed peak of HRSV cases in South America [38], suggesting that this seasonality may be correlated with lineage dynamics.

Contrary to A.D.3.3, we indeed expected to find the HRSV‐B lineage B.D.E.1 as the most common one in our data. This lineage comprises 19.5% of the sequences deposited in the Pathoplexus database and, during the period encompassed by this work, B.D.E.1 was at least 70% of the monthly HRSV‐B submissions in the GISAID database. This monolithic presence is likely the product of the population bottleneck that HRSV‐B suffered in 2021 from the mitigation measures against the COVID‐19 pandemic [3, 39]. Still, multiple differentiated clades within B.D.E.1 can be seen in our phylogenetic tree (Figure 2), indicating possible undescribed sublineages. Masking this hidden diversity under a single lineage designation may obscure epidemiological patterns relevant for disease prevention and control. For example, these clades can harbor new mutations with the potential to affect viral fitness, antigenicity, or the sensitivity of diagnostic assays.

Our analyses also uncovered an extensive list of viral co‐detections, including between HRSV‐A and HRSV‐B, even though we used a conservative threshold to consider a positive case as one. Coinfections with HRSV appear to be common, and there is extensive literature describing multiple cases. For example, analysis of a pediatric cohort revealed that 36% of HRSV‐infected patients had a second respiratory viral infection and an additional 19% had at least two more infecting viruses [40]. There is an intense discussion about whether HRSV coinfections aggravate diseases, but there is no consensus [2, 3]. On the other hand, there is strong evidence demonstrating that coinfections with bacteria, especially *Streptococcus*, have a significant impact on hospitalization, intensive care unit admission, and death [41]. Importantly, our results demonstrated that genomic analyses enhance the detection of coinfections beyond specific tests and can be a powerful diagnostic tool in hospital settings.

Finally, our data also reinforce the vulnerability of small children to HRSV in Brazil, with more than 75% of infections happening in infants up to a year old. Estimates in Brazil indicate that this age group composes from ~57% to 73% of all HRSV‐associated respiratory infections [42, 43, 44], values higher than the expected rate of 55% calculated from global data [45]. A higher number of HRSV‐related hospitalizations and deaths in children has been repeatedly reported in low‐ and middle‐income countries when compared to high‐income ones [5, 46]. While the exact reasons for such disparity have not been elucidated, factors associated with higher risk of HRSV infections (e.g., low birth weight and prematurity) are more accentuated in low‐ and middle‐income countries [47]. To reduce the incidence of HRSV in low/middle‐income countries, it is of utmost importance that maternal vaccination and the use of monoclonal antibodies in children under 2 years are incorporated into public health protocols, as Brazil has recently done.

It is possible, though, that the higher percentage of small children in our positive cases is due to undersampling people older than 60 years. The only adults positive for HRSV in our dataset were those investigated due to being critically ill, while in the surveillance program all infected patients were children. In developed countries, elders can represent up to 31% of hospitalizations [48], while in Brazil the proportion of senior people among the infected was estimated to range from 1.6% to 5.7% [43, 44, 49]. Conceivably, the surveillance programs in Brazil may be failing to capture the senior cohort infected by HRSV. Staadegaard and collaborators (2021) also suggested that lower viral titers and atypical symptoms could be the cause of the lower number of infected elders, but reluctance to seek treatment and poor access to public healthcare should also be considered [50]. Understanding the factors at play in HRSV‐related hospitalizations of elderly people is of urgent importance, given the higher mortality observed in this age group [49, 51].

It is important to reinforce that all the analyses conducted here can be strongly affected by sampling biases. The steps of reconstructing ancestor locations in the phylodynamic analyses are particularly susceptible to this, which could result in intermediate locations missing from transmission routes. Despite the high toll on public healthcare, molecular sequencing of HRSV is still rarely conducted in Brazil, and there were no genomic sequences from the country in the Pathoplexus database that could be used to enrich our analysis and avoid bias in our sampling (with the exception of a single genome without finer location information).

## 5 Conclusion

Our study provides a comprehensive phylodynamic portrait of HRSV circulation in the state of Rio de Janeiro, revealing a scenario shaped by repeated viral introductions, strong spatial structuring centered around the capital, and the emergence and expansion of lineages with potential epidemiological relevance. The prominent role of the city of Rio de Janeiro as a dispersal hub underscores the dynamic nature of HRSV spread in a highly connected setting. At the same time, the presence of frequent coinfections and the likely underrepresentation of elderly cases highlight critical gaps in our understanding of HRSV epidemiology in Brazil. Our findings strongly advocate for the integration of targeted genomic surveillance strategies within the Brazilian public healthcare system, as expanding sequencing efforts and improving representativeness will be essential to propose effective public health interventions against HRSV.

## Author Contributions

Study conceptualization and funding acquisition: Ana Tereza Ribeiro Vasconcelos; project administration: Ana Tereza Ribeiro Vasconcelos and Claudia Maria Braga de Mello; supervision: Ana Tereza Ribeiro Vasconcelos and Andréa Cony Cavalcanti; investigation: Alessandra Pavan Lamarca, Ana Tereza Ribeiro Vasconcelos, Melise Chaves Silveira, Renata Campos Azevedo; resources, including sample preparation and sequencing: Adriana Cardoso Camargo, Alexandra Lehmkuhl Gerber, Ana Paula C. Guimarães, Andréa Cony Cavalcanti, Leandro Magalhães de Souza; data curation: Adriana Cardoso Camargo, Andréa Cony Cavalcanti, Melise Chaves Silveira, Leandro Magalhães de Souza, Renata Campos Azevedo; methodology: Adriana Cardoso Camargo, Alessandra Pavan Lamarca, Andréa Cony Cavalcanti, Maria Angélica Arpon Marandino Guimarães, Melise Chaves Silveira, Leandro Magalhães de Souza; formal analysis: Alessandra Pavan Lamarca, Melise Chaves Silveira; visualization: Alessandra Pavan Lamarca; writing original draft: Alessandra Pavan Lamarca and Renata Campos Azevedo; reviewing and editing: Alessandra Pavan Lamarca, Ana Tereza Ribeiro Vasconcelos, Maria Angélica Arpon Marandino Guimarães, Melise Chaves Silveira, Renata Campos Azevedo. All authors read and approved the final manuscript.

## Ethics Statement

The present work was approved by the Ethics Committee of Universidade Federal do Rio de Janeiro (Process number 7.686.303).

## Conflicts of Interest

The authors declare no conflicts of interest.

## Supporting information


Supporting File 1

Supporting File 2

Supporting File 3

Supporting File 4

## Data Availability

The sequences reported in this paper have been deposited in the GenBank database (accession nos. listed in Table S1).
